# Hydrate of Chloral

**Published:** 1880-03

**Authors:** 


### HYDRATE OF CHLORAL.

Dr. H. H. Kane, of New York City, specially requests members of the profession with any experience whatever in the use of the Hydrate of Chloral to answer the following questions, and give any information they may possess with reference to the literature of the subject:

1. What is your usual commencing dose?
2. What is the largest amount you have administered at one dose, and the largest amount in twenty-four hours?
3. In what diseases have you used it (by the mouth, rectum, or hypodermically), and with what results?
4. Have you known it to affect the sight?
5. Have you ever seen cutaneous eruptions produced by it?
6. Do you know of any instances where death resulted from or was attributed to its use? If so, please give full particulars as to disease for which given; condition of pulse, pupils, respiration and *temperature*; manner of death; condition of heart, lungs and kidneys; general condition, age, temperament, employment, etc., etc., etc. If an autopsy was held, please state the condition there found.
7. Have you seen any particular manifestations from chloral—as tetanus, convulsions, or delirium?
8. Do you know of any cases of the chloral-habit? If so, please state the amount used, the disease for which the drug was originally administered, the person's temperament, and the present condition of the patient.

Physicians are earnestly requested to answer the above questions fully, especially 6 and 8, in order that the resulting statistics may be as valuable as possible.

All communications will be considered strictly confidential, the writer's name not being used when a request to that effect is made. Address all letters to Dr. H. H. Kane, 191 West 10th Street, New York City.

